# Antibacterial nanoagents: an emerging arsenal against bacterial persisters

**DOI:** 10.3389/fmicb.2025.1688413

**Published:** 2025-10-07

**Authors:** Yanling Hu, Dongliang Yang, Lihui Yuwen, Guisheng Zeng

**Affiliations:** ^1^College of Life and Health, Nanjing Polytechnic Institute, Nanjing, China; ^2^Key Laboratory of Flexible Electronics (KLOFE) and Institute of Advanced Materials (IAM), School of Physical and Mathematical Sciences, Nanjing Tech University (NanjingTech), Nanjing, China; ^3^State Key Laboratory of Flexible Electronics (LoFE) & Jiangsu Key Laboratory for Biosensors, Institute of Advanced Materials (IAM), Nanjing University of Posts & Telecommunications, Nanjing, China; ^4^A*STAR Infectious Diseases Labs (A*STAR ID Labs), Agency for Science, Technology and Research (A*STAR), Singapore, Singapore

**Keywords:** bacterial persisters, nanomaterial, recurrent infection, antimicrobial strategies, bacterial infection

## Abstract

Bacterial persisters represent a metabolically dormant or slow-growing subpopulation within bacterial communities that exhibit resistance to antibiotics. These cells are capable of resuming active proliferation upon the removal of environmental stressors, hence serving as reservoirs for recurrent infections. Extensive clinical evidence links persister cell formation with chronic infection and post-therapeutic recurrence. In this minireview, we highlight the challenges in eradicating persisters and review recent advances in nanomaterial-based antimicrobial strategies that specifically target these resilient cells. We also discuss key translational barriers impeding the clinical application of antibacterial nanoagents. By integrating these insights, we aim to provide a conceptual roadmap for the development of next-generation therapies against persistent bacterial infections.

## 1 Introduction

Bacterial infections have long posed a significant threat to global public health (Gollan et al., 2019; Zhang et al., 2023). The discovery of antibiotics had been one of the most significant breakthroughs in modern medicine, which dramatically reduced mortality from bacterial diseases. Today, antibiotics remain the cornerstone of bacterial infection treatment (Mo et al., 2025; Zhang et al., 2024b). However, widespread misuse and overuse of antibiotics have led to the growing crisis of antimicrobial resistance (Dai et al., 2012; Hu et al., 2025), rendering many once-reliable antibiotics increasingly ineffective (Zhang et al., 2024a). Alarmingly, drug-resistant infections are responsible for over 1.2 million deaths annually, a number projected to rise to 10 million by 2050, surpassing cancer-related mortality (Xu et al., 2025). Even when initial antibiotic treatment is successful, infection relapse remains a major clinical challenge. Such relapses prolong patient suffering, extend antibiotic treatments, and accelerate the emergence of resistant bacterial strains, fueling a vicious cycle that undermines current therapeutic approaches (Kushwaha et al., 2024).

Infection recurrence is mainly due to the formation of biofilms and the transition of bacterial cells into a dormant, antibiotic-tolerant state known as persistence (Fisher et al., 2017). Biofilms—structured microbial communities encased in a protective extracellular matrix—impede antibiotic penetration and shield bacteria from host immune defenses (Lv et al., 2023; Xiu et al., 2020; Yang et al., 2023). In response to environmental stress or survival threats, some bacterial subpopulations become metabolically inactive and stop replication (Yin et al., 2025). Unlike genetically acquired antibiotic resistance, persisters exhibit antibiotic tolerance by entering a metabolically quiescent state (Yin et al., 2025). Because conventional antibiotics target actively dividing bacteria by disrupting essential metabolic functions, persister cells evade killing during treatment (Ahmad et al., 2025). When antibiotic concentrations drop below therapeutic thresholds or the host immune response wanes, these dormant cells can resuscitate and proliferate, ultimately causing infection relapse (Sett et al., 2024). While prolonged, high-dose antibiotic regimens may offer temporary relief, such therapeutic approaches pose risks of promoting resistance and disturbing the host microbiota (Szajewska et al., 2025). Thus, devising targeted strategies to eliminate persister cells has become a pressing priority for improving clinical outcomes in bacterial infections (Fisher et al., 2017).

**Figure 1:**
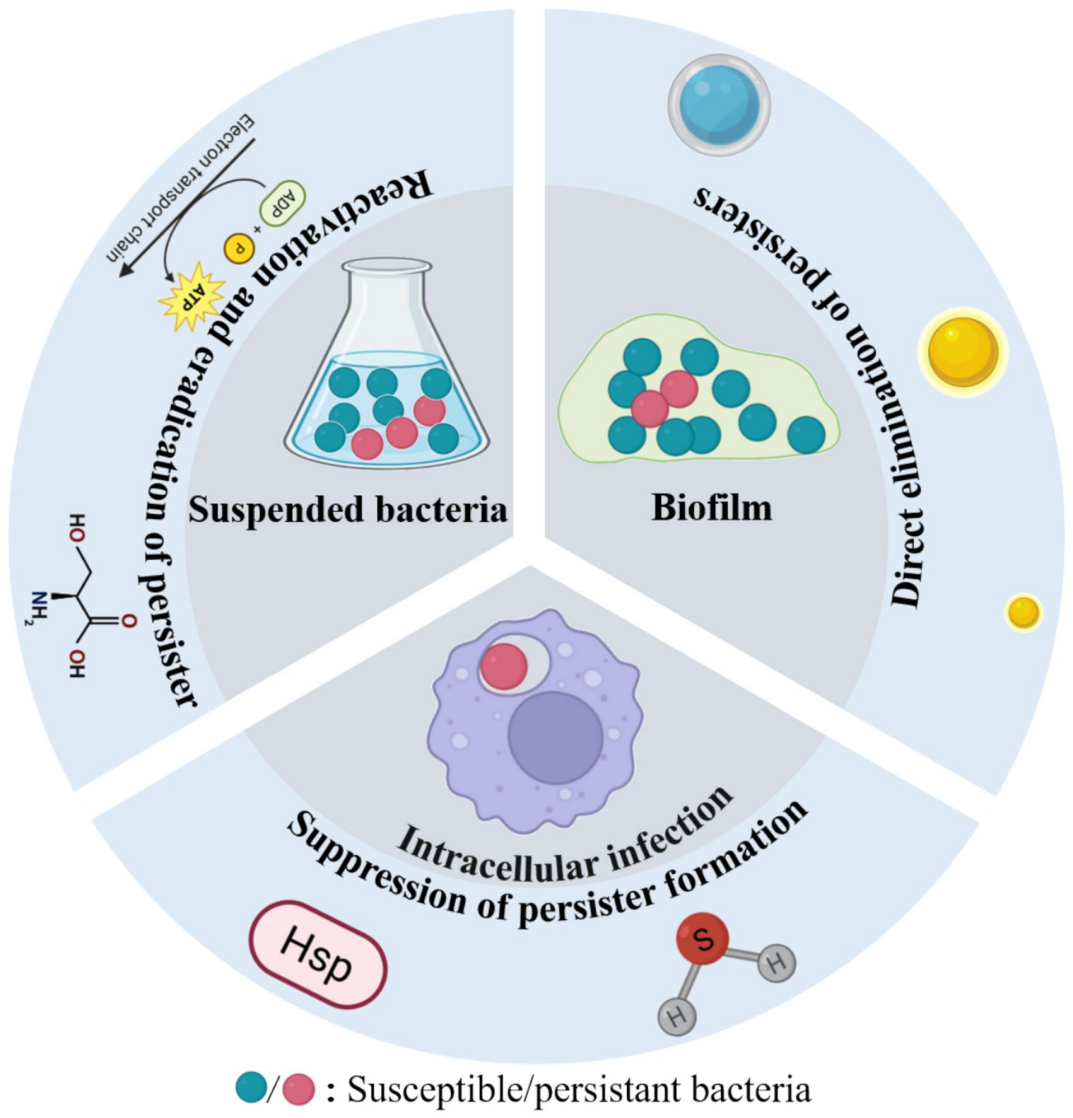
Nanoparticle-based antibacterial strategies for the eradication of persistent bacteria.

Antimicrobial nanomaterials combat bacterial persisters through diverse mechanisms, including physical disruption of cell structures, chemical interference with metabolic pathways, and biologically mediated effects (Geng et al., 2023; Zhang et al., 2024b). Compared to conventional antibiotics, these nanoagents exhibit superior efficacy against bacterial persisters due to several distinct advantages. First, their nanoscale dimensions and enhanced permeability enable deep penetration through dense extracellular polymeric substances (EPS), facilitating direct interaction with dormant cells embedded within biofilms. Second, their multimodal mechanisms of action—such as membrane perforation, reactive oxygen species (ROS) generation, and synergistic drug delivery—collectively reduce the likelihood of resistance development. Third, advanced surface functionalization enables biofilm matrix degradation, quorum sensing disruption, and targeted, sustained drug delivery and release, prolonging antimicrobial efficacy while minimizing adverse effects (Kalelkar et al., 2022; Makabenta et al., 2021). Notably, many of these materials exhibit excellent biocompatibility, making them promising candidates for clinical applications. In general, antibacterial nanoagents can effectively eliminate bacterial persisters through three principal strategies: (1) direct elimination of persisters, (2) reactivation of dormant bacteria followed by their eradication, and (3) suppression of persistent bacteria formation (Figure 1) (Yin et al., 2025). The following section summarizes the three aspects mentioned above and Table 1.

**Table 1:** Nanoagents for the removal of persistent bacteria.

Materials	Mechanism of action	Infection model	Ref
Caff-AuNPs	Direct elimination of persisters	*In vitro*, planktonic and biofilm-associated persisters	Khan et al. (2021)
AuNC@ATP	Direct elimination by enhanced bacterial membrane permeability and disrupted outer membrane protein folding	*In vitro*, planktonic persisters	Bekale et al. (2023)
AuNC@CPP	Direct elimination by disrupting the proton gradient across the membrane	Chronic suppurative otitis media	Cao et al. (2022)
MPDA/FeOOH-GOx@CaP	Direct elimination by ROS	Prosthetic joint infections	Chen et al. (2024)
PS^+^(triEG-*alt*-octyl)	Reactivation of persisters by activating the electron transport chain proteins	*In vitro*, biofilm-associated persisters	Huang Y. et al. (2025)
FAlsBm	Reactivation of persisters by using serine	*S. aureus* persister-induced peritonitis model	Huang D. et al. (2025)
LM@PDA NPs	Suppression of persister formation by neutralizing H_2_S	Persister- and biofilm-associated catheter infections	Hou et al. (2024)
Ti_3_C_2_	Suppression of persister formation by stimulating HSP expression	Dental caries	Zhang Y. et al. (2025)

## 2 Literature search method

To identify relevant references, we performed a systematic literature search using Google Scholar. The primary keyword pairs used were “bacterial persister” AND “nano” or “persistent bacteria” AND “nano.”

## 3 The recent treatment strategies for Persistent bacterial infections

### 3.1 Direct elimination of persisters

Effective elimination of persistent cells requires potent disruption of essential bacterial components, including membranes, proteins, and nucleic acids. Recent researches highlight the potential of novel antibiotics (e.g., polymyxin B, colistin, and aureomycin A), antimicrobial peptides, and metal ions in achieving this goal (Niu et al., 2024). These agents exert strong bactericidal effects even against dormant populations, offering promising avenues for treating persistent infections. However, its potential side effects may prevent its use in living organisms.

Nanomaterials have garnered increasing attention in this context due to their facile functionalization, controllable release kinetics, and low toxicity (Lu et al., 2022; Zuo et al., 2026). For instance, Khan *et al*. developed caffeine-functionalized gold nanoparticles (Caff-AuNPs), which exhibited potent bactericidal activity against both planktonic and biofilm-associated Gram-positive and Gram-negative bacterial persisters. These nanoparticles not only disrupted mature biofilms but also effectively eradicated embedded dormant cells (Khan et al., 2021).

Bekale and colleagues further advanced this approach by engineering adenosine triphosphate (ATP)-functionalized gold nanoclusters (AuNC@ATP) that selectively enhanced bacterial membrane permeability and disrupted outer membrane protein folding. Remarkably, these nanoclusters achieved a dramatic 7-log reduction in persister cell populations at a concentration of 2.2 μM, while exhibiting minimal toxicity toward exponentially growing bacteria (<1 log reduction) (Bekale et al., 2023). Subsequently, the same group developed nanoclusters modified with a cell-penetrating peptide (CPP, amino acid sequence: YGRKKRRQRRRCPP), termed (AuNC@CPP). These CPP-modified nanoclusters induced membrane hyperpolarization by disrupting the proton gradient across the membrane. When combined with the antibiotic ofloxacin, AuNC@CPP effectively eradicated *Pseudomonas aeruginosa* PA01 persisters, demonstrating strong therapeutic potential for chronic suppurative otitis media (Cao et al., 2022).

**Figure 2:**
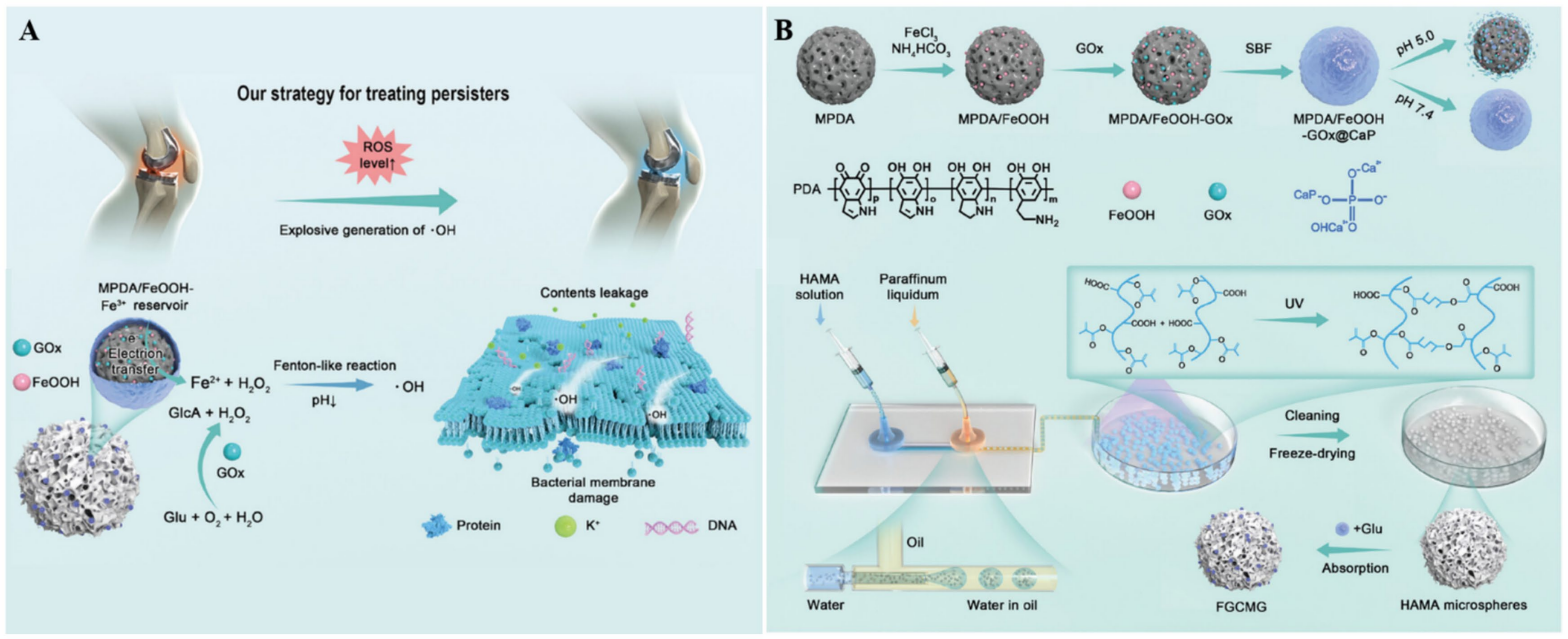
**(A)** The therapeutic mechanism of HAMA microspheres toward chronic periprosthetic joint infection. **(B)** The preparation process of HAMA microspheres. Reproduced with permission (Chen et al., 2024). Copyright 2024, Wiley.

Bacterial persisters are a key pathogenic factor in the recurrence of both chronic suppurative otitis media and prosthetic joint infections. To address this clinical challenge, Chen’s group developed ROS-generating hydrogel microspheres (MPDA/FeOOH-GOx@CaP) through a sophisticated multistep fabrication process (Figure 2) (Chen et al., 2024). First, the hydroxy iron oxide (FeOOH) nanocatalysts were grown *in situ* on mesoporous polydopamine (MPDA), followed by loading of glucose oxidase (GOx), The resulting nanoparticles were then sealed with a calcium phosphate (CaP) coating. Using microfluidic technology, the team co-encapsulated MPDA/FeOOH-GOx@CaP and glucose within hyaluronic acid methacrylate microspheres to form the HAMA composite gel microspheres. In the acidic microenvironment of infection sites, CaP dissolves, releasing GOx to catalyze the oxidation of glucose into H_2_O_2_, which is subsequently converted by FeOOH into membrane-damaging hydroxyl radicals via Fenton-like reactions. These hydrogel microspheres effectively eradicated *Staphylococcus aureus* and *Staphylococcus epidermidis* persisters, offering a promising strategy for treating prosthetic joint infections.

### 3.2 Reactivation of dormant bacteria followed by their eradication

**Figure 3:**
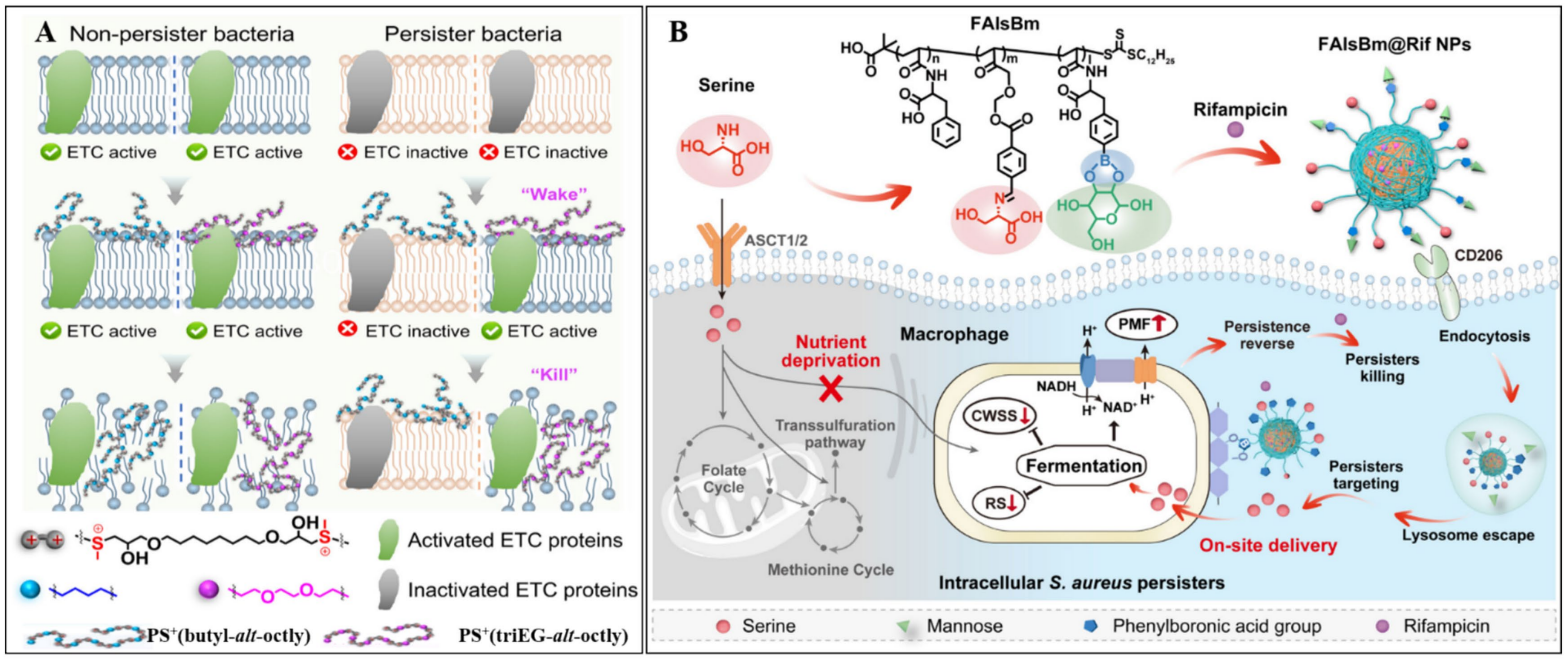
**(A)** The principle of PS^+^(triEG-*alt*-octyl) polymer in removing persister bacteria. Reproduced with permission (Huang Y. et al., 2025). Copyright 2025, American Chemical Society. **(B)** Mechanism of FalsBm@Rif NPs in eliminating intracellular *Staphylococcus aureus* persister. CWSS, cell wall stress stimulation; SR, stringent response. Reproduced with permission (Huang D. et al., 2025). Copyright 2025, American Chemical Society.

Metabolic activation strategies have become a widely used approach for eliminating bacterial persisters. Recent studies have demonstrated that stimulating the electron transport chain can effectively reactivate dormant bacteria, rendering them susceptible to antimicrobial agents (Kim et al., 2016). Building on this principle, Huang Y. et al. (2025) developed a cationic polymer, PS^+^(triEG-*alt*-octyl), capable of “waking up and killing” persisters (Figure 3A). This polymer first activates electron transport chain proteins, reawakening dormant bacteria, and subsequently disrupts bacterial membranes, leading to cell lysis and death. To improve delivery efficiency within bacterial biofilms, PS^+^(triEG-*alt*-octyl) was loaded onto PDA nanoparticles (triEG referring to triethylene glycol segment). Upon light irradiation, the PS^+^(triEG-*alt*-octyl)_PDA_ nanoparticles enable photothermal-triggered polymer release. In addition, PDA enhances the diffusion across the biofilm’s EPS. As a result, the PS^+^(triEG-*alt*-octyl)_PDA_ nanoparticles exhibit potent antibiofilm activity, effectively clearing persistent biofilms.

In a parallel study, Wang et al. (2021) developed a poly-amino acid-based nanodelivery system (FAlsBm) by covalently conjugating mannose (Man) and serine (Ser) to the side chains of a polymer [i.e., poly(N-acryloyl phenylalanine)-b-poly(2-((4-formylbenzyl)oxy) acrylic acid ethyl ester)-b-poly((S)-2-acrylamido-3-(4-boronphenyl) propionic acid, FAlsB) via ester and imine linkages]. Rifampicin (Rif) was then encapsulated through self-assembly (Figure 3B) (Huang D. et al., 2025). The resulting FAlsBm@Rif system featured a dual-targeting mechanism: first, mannose-mediated macrophage-specific uptake via CD206 mannose receptor binding; second, upon lysosomal internalization, acidic cleavage of ester/imine bonds released Man/Ser while exposing phenylboronic acid groups, which redirected the nanoparticles to *Staphylococcus aureus*. The liberated serine acted as a nutritional stimulus to revive dormant bacteria, enhancing their susceptibility to rifampicin. In animal experiments, FAlsBm@Rif, achieved 99.78% clearance of intracellular bacteria—a 1.6-fold improvement over conventional rifampicin treatment (63.41% clearance). This intelligent “cell-to-bacteria” targeting strategy offers a promising solution for combating recalcitrant intracellular infections.

### 3.3 Suppression of persistent bacteria formation

The formation of bacterial persisters is a multifactorial process regulated by several key regulatory mechanisms, including toxin-antitoxin (TA) systems, stress response pathways mediated by (p)ppGpp signaling, SOS repair systems, and quorum sensing (Niu et al., 2024; Yin et al., 2025). Recent studies have suggested that hydrogen sulfide (H_2_S) and heat shock proteins (HSPs) are also involved in this process. Under environmental stress, bacteria produce H_2_S, which inhibits the synthesis of adenosine triphosphoric acid, induces a dormant state, and promotes the formation of drug-resistant persister cells. To counteract this mechanism, Hou et al. (2024) developed an innovative H_2_S-scavenging antibacterial platform. Using ultrasonication, they synthesized PDA-coated liquid metal (LM) gallium nanoparticles (LM@PDA NPs), which were subsequently functionalized with urease and silver nanoparticles (Ag NPs) to produce a multifunctional nanocomposite (LPUA NPs). The PDA coating ensures stable immobilization of LPUA NPs onto urinary catheter surfaces, where they exhibit dual biological functions. First, urease catalyzes the hydrolysis of urea to generate ammonia, which neutralizes bacterial H_2_S. In parallel, excess H_2_S reacts with liquid gallium to form Ga_2_S_3_, thereby suppressing persister cell formation and enhancing the antibacterial activity of released Ga^3+^/Ag^+^. Second, the intrinsic deformability of LM nanoparticles facilitates deep biofilm penetration, enabling efficient bacterial clearance.

**Figure 4:**
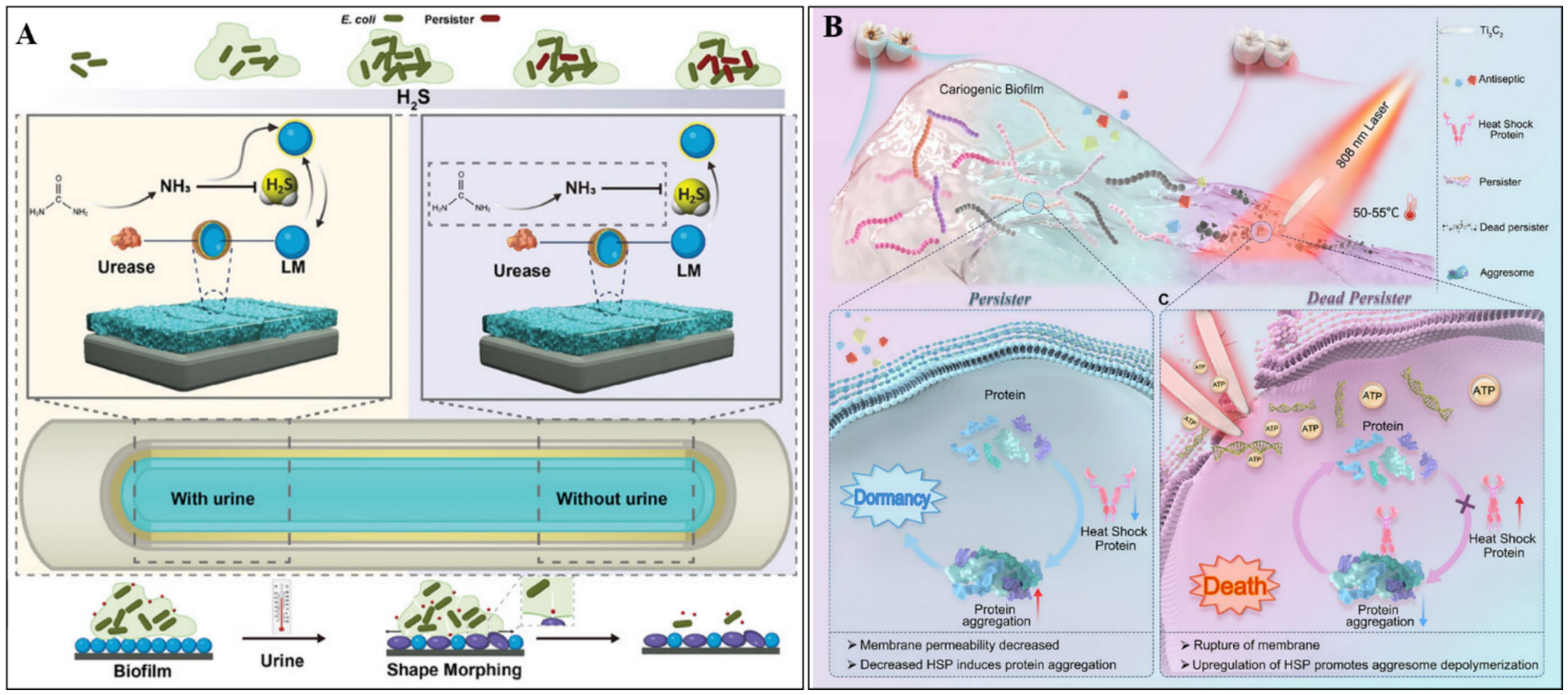
**(A)** The inhibitory mechanism of LM@PDA@Urease&Ag NPs (LPUA NPs) against persistent bacterial formation. Reproduced with permission (Hou et al., 2024). Copyright 2024, Wiley. **(B)** Ti_3_C_2_-based photothermal therapy suppresses persistent bacterial formation via inducing HSP expression and eradicates persister cells by nanoknife effect and photothermal removal. Reproduced with permission (Zhang Y. et al., 2025). Copyright 2025, Wiley.

Adverse environmental conditions can induce protein misfolding in bacteria, leading to protein aggregation, metabolic activity slowdown, and persister cell formation. HSPs play a critical role in maintaining proteostasis by preventing protein aggregation. Leveraging the temperature-dependent upregulation of HSP expression, Zhang Y. et al. (2025) employed the photothermal and nanoknife properties of Ti_3_C_2_ MXene to suppress persister formation and enhance the elimination of cariogenic bacteria. Upon light irradiation, Ti_3_C_2_ generates localized heat, stimulating HSP expression to mitigate protein misfolding, thereby reducing persister formation and increasing bacterial membrane permeability. Simultaneously, Ti_3_C_2_-mediated photothermal effect disrupts bacterial membranes, causing cytoplasmic leakage and cell death. Compared to traditional antibiotics, this strategy exhibits superior efficacy in eradicating both planktonic and biofilm-embedded persister cells, offering a promising therapeutic avenue for the treatment and prevention of dental caries (Zhang Y. et al., 2025) (see Figure 4).

## 4 Conclusion and outlook

In conclusion, this minireview outlines the challenges of eliminating persistent bacteria and the potential of nanoantibacterial agents to address them. Then these nanoagents are systematically categorized by their mechanism of action: direct eradication, awakening persistent bacteria, or inhibiting persistent bacteria formation. Despite their promise, these approaches face several challenges that must be resolved before broader application.

Antibacterial nanomaterials are currently synthesized using a range of methodologies, including physical techniques (e.g., laser ablation, ball milling), chemical approaches (e.g., chemical reduction, sol–gel), and bio-based strategies (e.g., plant- or microorganism-mediated green synthesis) (Rathore et al., 2023; Song et al., 2026; Wei et al., 2023). Their development is increasingly oriented toward green sustainability, precise synthesis control, and scalable industrialization. In clinical contexts, these antibacterial nanoagents demonstrate significant potential across various applications, including anti-biofilm therapies (e.g., medical device coatings, chronic wound management), targeted drug delivery systems (e.g., antibiotic-loaded nano-carriers), and integrated diagnostics-therapeutics (e.g., multimodal imaging-guided antibacterial interventions). However, clinical translation remains limited due to several key challenges: (1) Biological safety concerns, such as nanoparticle-induced cytotoxicity and long-term biodistribution risks; (2) Evolution of antibacterial resistance; (3) Manufacturing challenges, including batch-to-batch variability, nanoparticle aggregation, and issues with the stability of antibacterial activity of material properties during storage (Solanki et al., 2024; Zhang J. et al., 2025); (4) Lack of standardized international regulatory frameworks for nanomedicine evaluation and approval; (5) Current research lacks a deep understanding of how nanomaterials specifically target and eliminate persistent bacteria. Consequently, a key challenge is the precise design of nanoscale antibacterial agents to enhance their targeting and efficacy while minimizing toxicity to host cells.

To overcome these barriers, several innovative strategies are being explored: (1) Toxicity mitigation through surface functionalization (e.g., PEGylation) and the use of biodegradable matrices (e.g., chitosan); (2) Synergistic antibacterial regimens that integrate photothermal or photocatalytic therapies with conventional antibiotics to suppress resistance development (Cui et al., 2024; Wang et al., 2021); (3) Microfluidic-based precision synthesis to improve nanoparticle uniformity; (4) Promotion of harmonized international regulatory frameworks for clinic translation of nanomedicines; (5) Functionalize nanomaterials by targeting specific molecular markers unique to persistent bacteria (Zhang et al., 2021); (6) Design smart nanosystems that remain inert until activated by the unique signals of an infection site. Looking ahead, convergent technological innovations—such as AI-driven material optimization, organ-on-a-chip platforms for toxicity screening, and patient-specific nanotherapeutic designs—are poised to revolutionize the field of nanomedicine. These advancements may establish nanoscale antibacterial agents as pivotal tools in the post-antibiotic era, offering promising solutions to the growing threat of multidrug-resistant pathogens.

In addition, two critical challenges remain in the study of bacterial persisters: (1) The lack of standardized, high-throughput detection methods, and (2) Significant limitations in current screening approaches. Although antibiotic treatment is widely recognized as the primary trigger for persister cell formation, substantial variability in the metabolic profiles of these antibiotic-induced persisters remains—an observation that continues to generate scientific debate. Therefore, the establishment of reproducible induction protocols and the development of reliable, high-sensitivity detection methods are essential prerequisites for the rational design and evaluation of nano-therapeutics targeting persister cells.
